# Towards improving diagnosis of skin diseases by combining deep neural network and human knowledge

**DOI:** 10.1186/s12911-018-0631-9

**Published:** 2018-07-23

**Authors:** Xinyuan Zhang, Shiqi Wang, Jie Liu, Cui Tao

**Affiliations:** 1 School of Biomedical Informatics, The University of Texas Health Science Center at Houston, Huston, TX USA; 2 Department of Dermatology, Peking Union Medical College Hospital, Chinese Academy of Medical Sciences and Peking Union Medical College, Beijing, China

**Keywords:** Deep learning, Semantic data analytics, Image classification, Dermatology

## Abstract

**Background:**

The emergence of the deep convolutional neural network (CNN) greatly improves the quality of computer-aided supporting systems. However, due to the challenges of generating reliable and timely results, clinical adoption of computer-aided diagnosis systems is still limited. Recent informatics research indicates that machine learning algorithms need to be combined with sufficient clinical expertise in order to achieve an optimal result.

**Methods:**

In this research, we used deep learning algorithms to help diagnose four common cutaneous diseases based on dermoscopic images. In order to facilitate decision-making and improve the accuracy of our algorithm, we summarized classification/diagnosis scenarios based on domain expert knowledge and semantically represented them in a hierarchical structure.

**Results:**

Our algorithm achieved an accuracy of 87.25 ± 2.24% in our test dataset with 1067 images. The semantic summarization of diagnosis scenarios can help further improve the algorithm to facilitate future computer-aided decision support.

**Conclusions:**

In this paper, we applied deep neural network algorithm to classify dermoscopic images of four common skin diseases and archived promising results. Based on the results, we further summarized the diagnosis/classification scenarios, which reflect the importance of combining the efforts of both human expertise and computer algorithms in dermatologic diagnoses.

## Background

In biomedical informatics field, research has been done on using image-based artificial intelligence diagnosis system to help early detection of certain diseases, especially skin diseases [1, 2]. For pattern recognition and classification of clinical image, deep neural networks have been widely used. A recently published article in Nature provided an example for using a convolutional neural network (CNN) to disaggregate 2032 different kinds of skin diseases and tested its performance against 21 board-certified dermatologists [2]. The CNN performed on par with the experts, proving the feasibility of computer-aided diagnosis system. In the healthcare field, this system can potentially help healthcare providers make more effective diagnoses as a clinical decision support tool. There are multiple types of deep neural networks, including convolutional and recursive neural networks (CNN and RNN), etc. Some studies used deep neural networks to develop and modify image classification techniques [3–7]. Many previous studies showed promising results for both the reliability and accuracy of computer-aided decision support [8–10]. A subset of published articles examined the combination of human expertise and artificial intelligence [11, 12]. Nevertheless, we still need to better integrate human knowledge into artificial intelligence and to use artificial intelligence to extend human intelligence.

In our previous study, we classified four common cutaneous diseases based on dermoscopic images using deep learning algorithms [13]. This paper extended it by summarizing classification/diagnosis scenarios and semantically represented them in a hierarchical structure in hope to further facilitate decision-making and improve the accuracy of our algorithm. Four frequently seen skin diseases were selected for the study, melanocytic nevus, seborrheic keratosis (SK), basal cell carcinoma (BCC) and psoriasis. Melanocytic nevus is a very common benign cutaneous tumor. It can occur from infancy to old age, and the amount each person usually carries increases with age [14]. Seborrheic keratosis (SK), also known as basal cell papilloma, is due to the delayed maturation of benign epidermal cells. Melanocytic nevus and basal cell carcinoma both have deeper lesions, which must be accurately distinguished [15]. Basal cell carcinomas (BCC) is one of the most common human skin cancers, especially in the elderly. According to epidemiological studies, the incidence of BCC is increasing year by year, and the incidence in young people shows a gradual upward trend [16]. Psoriasis is a common and readily recurrent chronic inflammatory skin disease. Worldwide, the prevalence of psoriasis is 2–4%. It has a significant impact on the patient’s health and even mental health [17]. Therefore, psoriasis is always one of the foci of dermatological research.

These four cutaneous diseases: basal cell carcinoma (BCC), melanocytic nevus, seborrheic keratosis (SK), and psoriasis, are epidermal malignant, melanocytic benign, epidermal benign, and non-neoplastic skin diseases respectively. It is noteworthy that the treatment of these four diseases is very different. If the diagnosis is incorrect or the diagnosis is delayed, it may lead to improper treatment, treatment delay, even no treatment [18]. Therefore, it is critical that the providers can make accurate diagnosis in time. If the artificial intelligence system can be used to automatically classify these four diseases, providers can benefit patients by diagnosing them more efficiently and accurately.

## Methods

### Dataset

The data used in this study originated from the dermatology department of Peking Union Medical College Hospital. The clinical database currently contains more than 28,000 dermoscopic images examined by MoleMax HD 1.0 dermoscopic devices. Our study was approved by the Ethics Committee of Institute of Peking Union Medical College Hospital, Chinese Academy of Medical Sciences. Informed consent was obtained from all participants.

**Fig. 1:**
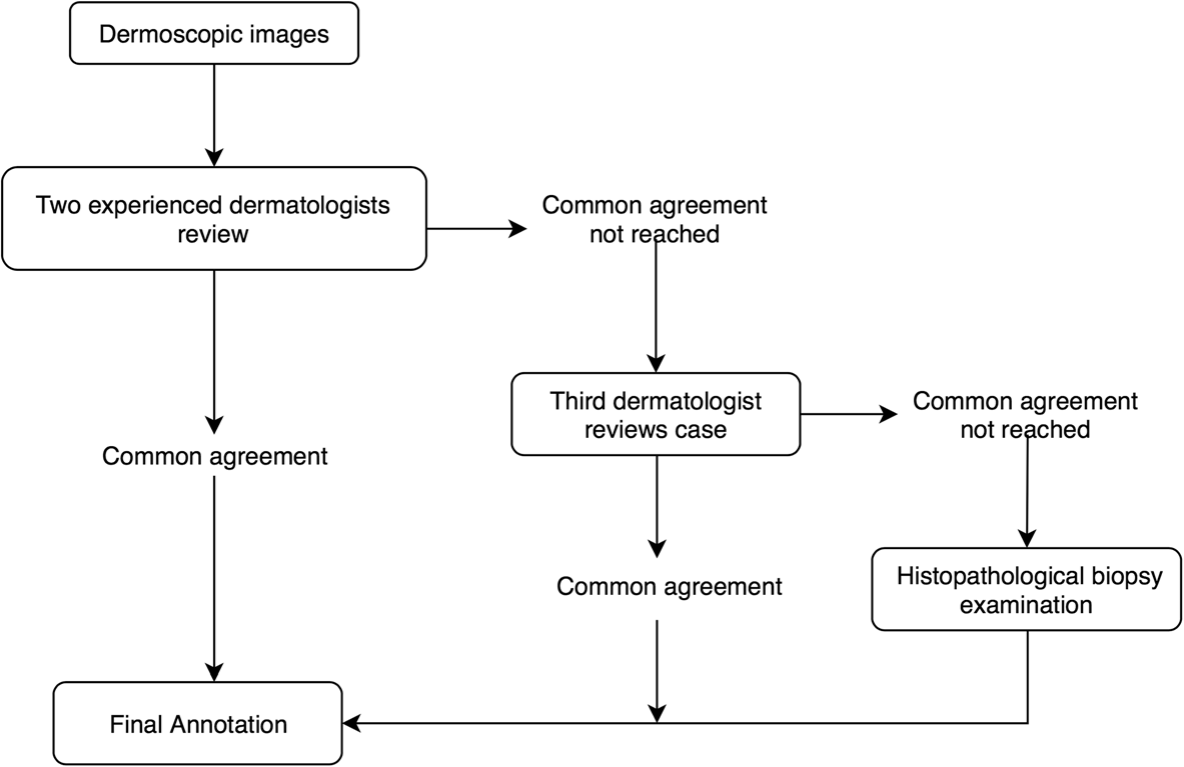
Procedure for annotating the dermoscopic image

**Fig. 2:**
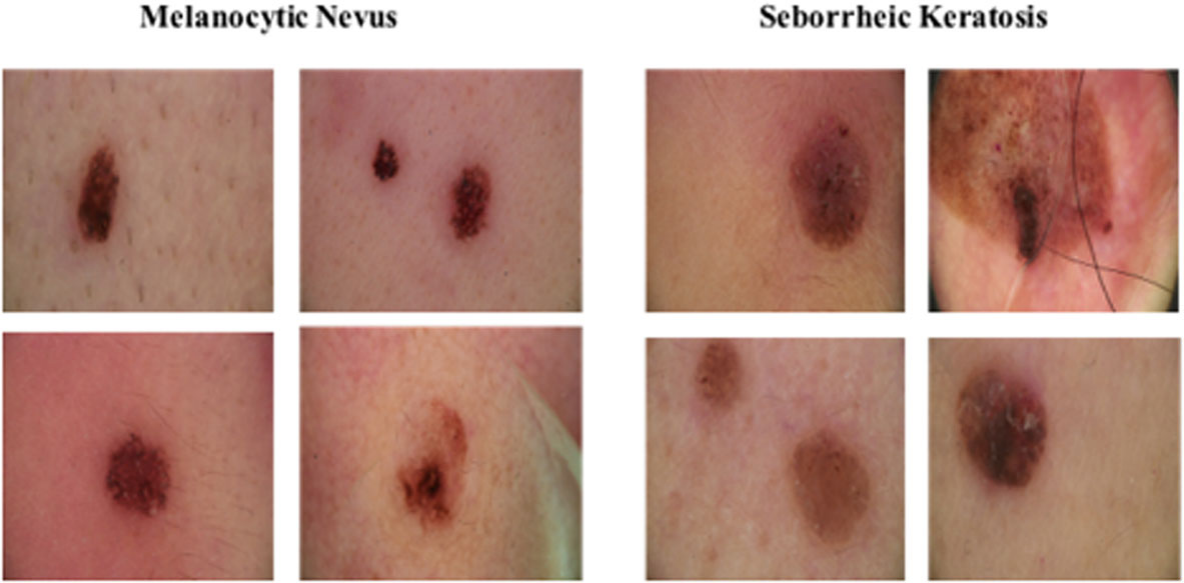
Example of typical dermoscopic images

Our experiment was developed based on a gold standard, where each image was rigorously reviewed by at least two experienced dermatologists before the diagnosis results were given. Figure 1 shows the annotation process. Figure 2 shows some examples of typical dermoscopic images. First, each dermoscopic image was reviewed by at least two experienced dermatologists. If consensus was reached, the resulting diagnosis was annotated. If not, a third dermatologist was consulted. If consensus was reached after discussion, the annotation reflected the agreed upon diagnosis. If not, the image was taken to the histopathological biopsy examination. In the end, each image was labeled with a disease name. As we have seen, this is a very time consuming and expensive process.

### Training image selection

All together, our experiment dataset contains 1067 images from patients who visited the clinic between 2015 and 2017 with images of 418 melanocytic nevus, 291 seborrheic keratosis, 132 basal cell carcinoma, and 226 psoriasis dermoscopic, respectively. We used two datasets in the experiment. Dataset A consists all the images. Dataset B was a selected, evenly distributed dataset. 132 images were selected from each disease category, for a total of 528 images. Details are listed in Table 1.

**Table 1:** Summary for datasets

Dataset A		Dataset B	
melanocytic nevus	418	melanocytic nevus	132
seborrheic keratosis (SK)	291	seborrheic keratosis (SK)	132
basal cell carcinoma (BCC)	132	basal cell carcinoma (BCC)	132
psoriasis	226	Psoriasis	132
Total number of images	1067	Total number of images	528

### Deep learning algorithm

**Fig. 3:**
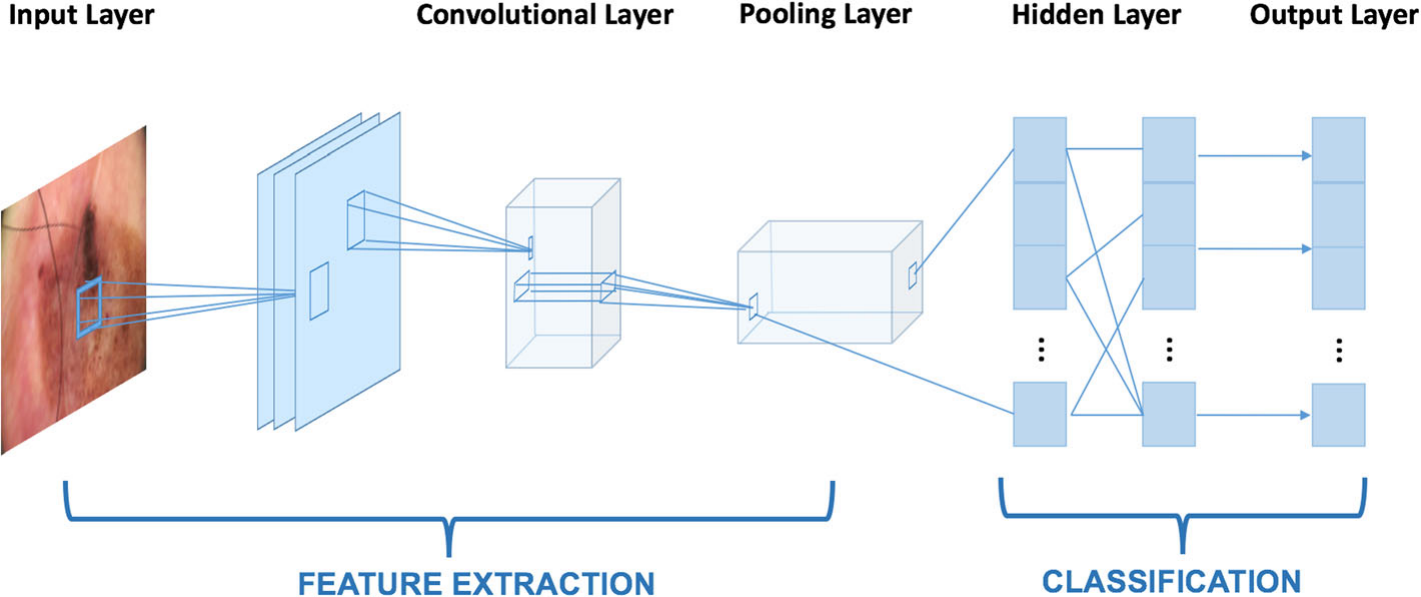
A Simplified framework for deep neural networks

We developed our algorithm based on GoogleNet Inception v3 code package which was pre-trained on over 1.28 million images [19]. We adjusted the final layer and used our datasets as input. The GoogleNet deep learning framework is a type of artificial neural network called CNN. It is inspired by a biological process called axonal transport or synaptic transmission, in which multiple neurons receiving signals partially overlap and cover the entire field [20]. CNNs have similar functions, where the calculated features are combined with each other. The simplified framework of the entire process is shown in Fig. 3. CNN is often used in real life for image or video recognition and natural language processing [21–23].

Each pixel in the input images was transformed into an element in matrices. For example, if an image has 864 pixels, multiplied by RGB layers, the number of elements in a matrix would be 2592. If there are 100 input images, the input matrix will have the dimension of 2592 * 100. This is also called the “Input Layer”. Each image then went through the feature extraction process, during which the combination of convolutional and pooling layers was used. A feature map was obtained by applying a linear filter and a non-linear function to the input matrix. For example, the hidden layer *A*^*k*^, where k is the *k*^*th*^ feature map. The filters here consisted of its weight *W*^*k*^ and its bias *b*_*k*_ . The feature map *A*^*k*^ was calculated using Eq. 1 [24]. Then the calculated feature map from extraction was classified. Each hidden layer was composed of multiple feature maps. There are multiple hidden layers used in the model.

1 $$ {\mathrm{A}}_{\mathrm{ij}}^{\mathrm{k}}=\kern0.5em \tanh \kern0.5em \left({\left({W}^k\kern0.5em \cdot \kern0.5em x\right)}_{ij}\kern0.5em +\kern0.5em {b}_k\right)\kern0.75em $$

The input layer is analogous to the receptive field of nerve cells. The hidden layer functions similarly to transmission down the axon, where the input signals are being processed. The terminal zone can be regarded as both the output layer and the receptive zone for the next cell, which is similar to the Inception 3 algorithm [19]. Data flows from left to the right. However, there is one difference between an artificial neural network and a biological one. Researchers developed backward error propagation to help tune the input activation functions, in which way the final layer of CNN was adjusted.

We ran the experiments using datasets A and B separately. Each data set was divided into training, validation and testing sets, in 8:1:1 ratio respectively. The prediction values were compared with the actual labels to update the final layer’s weights *W*^*k*^ [19], as shown in Eq. 1. The validation set was separated from the training set to avoid over-fitting [25]. The calculated parameters from the training set were examined using the validation set to see whether they fit as well, and recalculated as needed. This process can help the model to memorize less of the irrelevant or unimportant details of the training images. A hold-out testing set was used to evaluate the accuracy of the whole process. Each image appears only once in each set.

The classification results by the algorithm can be used for diagnoses. Each test image was given a probability for each of the four disease categories, summing to 1. The highest probability was regarded as the classification category. Healthcare providers will also be able to receive more detailed information, in addition to just one end result. These possibilities of the disease classifications can potentially be used to better perform semantic error analysis.

### Evaluation and semantic error analysis

We also conducted an error analysis on misclassified images using dataset B. In order to get enough data for systematic analysis and remain the uniqueness of testing set, we used ten-fold cross validation to repeat the algorithms ten times. The summarized report of all the misclassified dermoscopic images for dataset B was then reviewed by domain experts (SW and JL). A thorough analysis was then conducted based on which a semantic classification of the misclassified images was generated.

## Results

### Results for deep learning algorithm

To avoid random errors, we repeated the experiment procedure ten times to obtain data for validation. All the experiments are evaluated on different testing sets. The average accuracy and standard deviation for ten results are shown in Table 2 for datasets A and B.

**Table 2:** Summary for accuracy and standard deviation

	Avg. Accuracy	Standard deviation
Dataset A	87.25%	2.24%
Dataset B	86.63%	5.78%

To further evaluate this method, we also calculated precision, recall, and F1 score for each disease (Tables 3 and 4). In order to prevent potential bias from uneven distribution of four diseases, we used a balanced distribution, which is data set B. The formulas were as follows.

**Table 3:** Summary for Precision and Recall (Dataset B)

	BCC	melanocytic nevus	Psoriasis	SK
Precision	88.24%	89.06%	88.55%	79.07%
Recall	87.5%	88.37%	88.55%	80.31%
F-Measure	0.879	0.887	0.885	0.797

**Table 4:** Summary for classified Images (Dataset B)

Original annotation	Classified diseases			
	BCC	Melanocytic nevus	Psoriasis	SK
BCC	105	4	4	6
melanocytic nevus	4	114	1	9
Psoriasis	2	3	116	10
SK	9	8	10	102

2 $$ Precision\kern0.5em =\kern0.5em \frac{true\kern0.5em positive}{true\kern0.5em positive+\kern0.5em false\kern0.5em positive} $$

3 $$ \operatorname{Re} call\kern0.5em =\kern0.5em \frac{true\kern0.5em positive}{true\kern0.5em positive\kern0.5em +\kern0.5em false\kern0.5em negative} $$

4 $$ F- measure\kern0.5em =\kern0.5em 2\kern0.5em \cdot \kern0.5em \frac{precision\kern0.5em \cdot \kern0.5em recall}{precision\kern0.5em +\kern0.5em recall} $$

### Categories for misclassified images

**Fig. 4:**
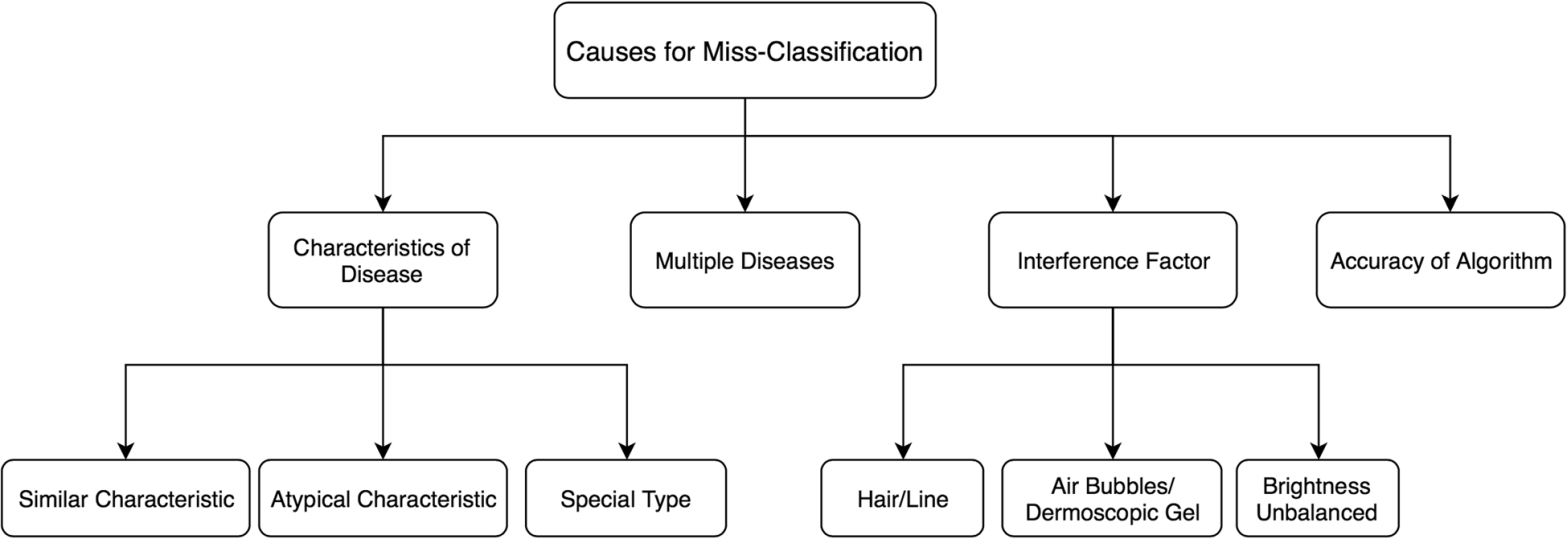
Knowledge Representation Tree for decision making

**Fig. 5:**
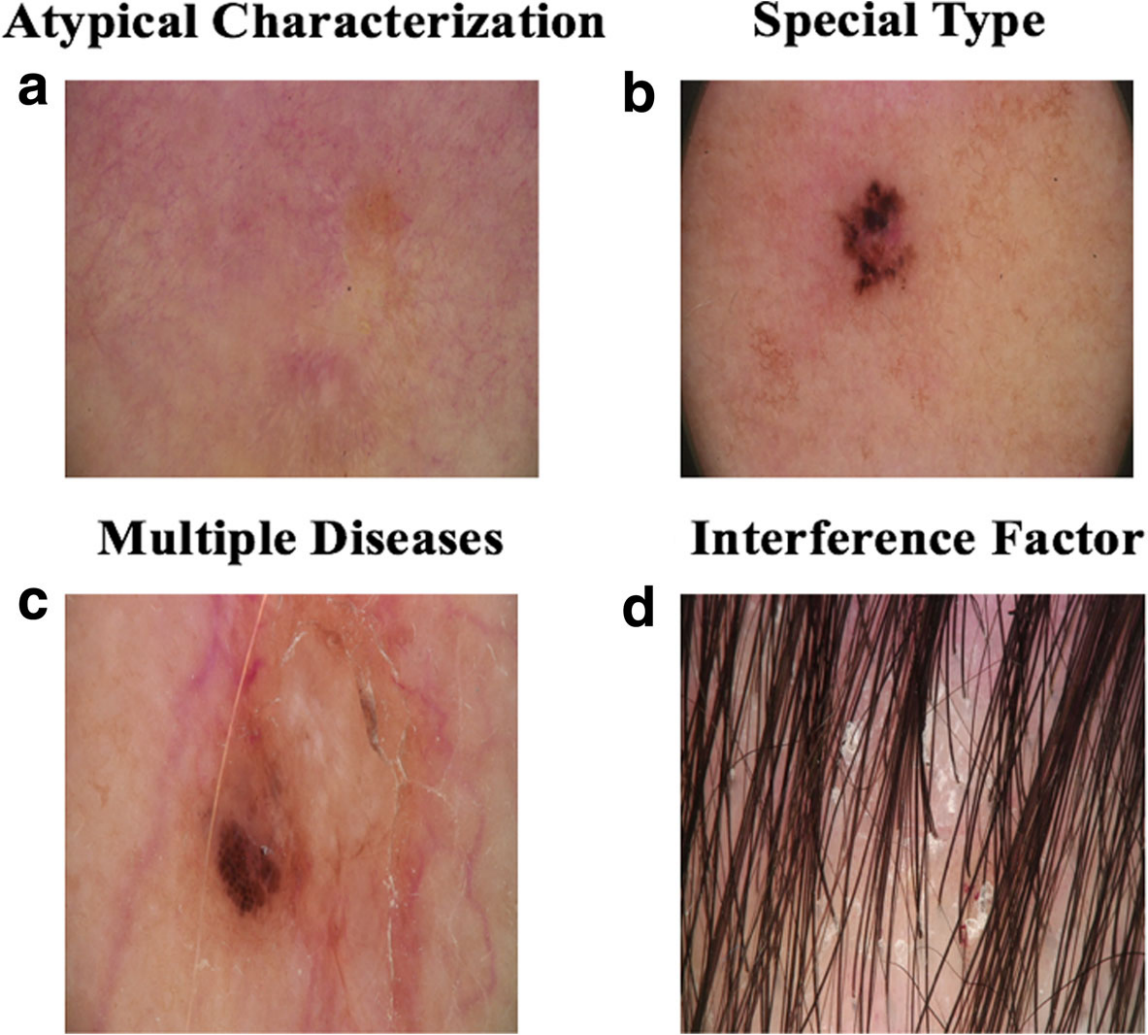
Examples of misclassified images under categories of﻿ possible error causes: **a** “Atypical Characterizatioin”, **b** “Special Type”, **c** “Multiple Diseases” and **d** “Interference Factor”

We then conducted an error analysis to summarize the reasons why certain images were misclassified by our algorithm. Two dermatologists and two informaticians reviewed these images and suggested possible causes for misclassifications. All the scenarios are summarized and represented using a hierarchical structure in Fig. 4. Figure 5 shows some examples of the misclassified cases.

As Fig. 4 shows, we have identified 4 major categories of possible error causes: “Characteristics of Disease”, “Multiple Diseases”, “Interference Factor” and “Accuracy of Algorithm”.

Under the category “Characteristics of Disease”, we further classified three subclasses: “Similar Characteristics”, “Atypical Characteristics” and “Special Type”. The category “Similar Characteristics” was defined to describe those errors caused by images that belong to different diseases but share similar features. These similar features could have affected the performance of the CNN classifier. The category “Atypical Characteristics” refers to those images that do not have typical features of the corresponding disease. For example, Fig. 5a is a regression lesion of psoriasis vulgaris. The typical features of the disease such as dotted blood vessels were significantly reduced, leaving only inflammation. Therefore, the misclassification belongs to “Atypical Characteristic”. The category “Special Type” defines the images that have abnormal features that usually do not appear in the corresponding disease. Figure 5b shows an example. This image was annotated as BCC but misclassified as melanocytic nevus. The image contains a large blue-gray oval nest and a leaf-like structure, but no branched blood vessels [17]. This case has many pigmented areas which covered most of the lesion. That may have been the cause of misdiagnosis.

The category “Multiple Diseases” refers to those misclassified images that contain lesions belonging to more than one disease. Image in Fig. 5c indicates both SK and BCC diseases and was classified by the algorithm to SK, but BCC lesions can also be seen in the image. The misclassification may be due to the fact that the pigmentation of SK is more obvious. In a subsequent study, we can preprocess the image to detect and separate multiple diseases.

“Hair/Line”, “Air Bubbles/ Dermoscopic Gel” and “Brightness Unbalanced” are all the misclassified causes under the “Interference Factor” category. These factors would interfere with our algorithm when identifying the critical attributes during classification. The image in Fig. 5d is psoriasis, but misclassified to SK. The image does contain the dermoscopic findings of the corresponding disease, but there is an interference factor, hair. Human eyes can usually avoid the interference factor without losing the features of the lesion, but CNN considers hair as a key factor for classification.

Our algorithm itself can be improved. There are some images that couldn’t be classified under any of the category above after reviewed by domain expert. Therefore, a category called “Accuracy of Algorithm” was added.

## Discussion

From Table 2, we can see that the average accuracy results are 87.25% for dataset A and 86.63% for dataset B. Since we only used a small portion of the images to train the algorithm, we believe our method is very promising. In addition, we can see that the results of dataset A and dataset B are not significantly different, so as a result, the deep learning algorithm can deal well with the unbalanced dataset. Moreover, the standard deviation of our results is about 2–5%, which can reflect the variation of accuracy. The relatively small number of images may be the reason.

The precision, recall, and f-measure values of SK are the lowest, while the other three diseases are all more than 87% on these measures (Table 3). As Table 4 shows, only 79.5% of the SK images were correctly classified. BCC with SK (15 images were misclassified) and melanocytic nevi with SK (17 images were misclassified) were often confused with each other. Previous studies also reported that they do have similar characteristics [26].

The categories of misclassification can facilitate future development of the system and help us to decide how to best incorporate human expertise. For some cases that our algorithm misclassified, it could be relatively easy for a human expert to make the right judgment. For example, for images with interference factors such as hair or air bubbles blocking the texture of skin lesions, human experts can often ignore the interfering factors and focus on the whole pattern, while a machine algorithm could accidently take those irrelevant factors as part of the features in training. There are techniques that can eliminate the hair factors automatically [27–29]. Nonetheless, the noise caused by the elimination process could generate more problems during automatic classification. Following the misclassification category, when an input picture was determined by the system under hair blocking category, we could attach it with semantic labels, such as the shape or edge of the lesions from the dermatologists to help with the classification.

Dermatologists will usually make a diagnosis based on more information than just the images themselves. For example, BCC occurs more commonly in elderly people [17], and high sun exposure can increase the risk of BCC in exposed body parts [30]. The occurrence of psoriasis has two peaks, one at age 20 to 30 and the second at age 50 to 60. In addition, it was more commonly seen in non-Hispanic whites [18]. These kind of prior knowledge need to be embedded in the algorithms in the form of semantic labels with additional patients’ data from EHR. These semantic features will incorporate more human knowledge to improve the accuracy of algorithm.

Our overarching goal is to develop a decision support system that can incorporate human knowledge into the process of artificial intelligence and then to use artificial intelligence to extend human capabilities. Our immediate next step is to improve our system by preprocessing the images and adding more semantic features. These improvements will be designed based on our defined semantic categories in Fig. 4. For example, we can modify the feature extraction computer algorithm to consider the atypical characteristics. If there were interference factor, we could attach the semantic labels as described before. Since we know the multiple diseases could be the reason for misclassification, preprocessing images to separate different lesions will be included in the model.

In addition, we will expand our dataset to test whether a larger dataset would result in better performance. We also plan to include more diseases and more types of images, such as histological images, smartphone images, etc. The ultimate goal is to deliver a decision support system to help clinicians make better diagnostic decisions and also create a patient-usable system which anyone can use with their mobile apps.

## Conclusion

In this paper, we applied deep neural network algorithm to classify dermoscopic images of four common skin diseases. Dataset A (1067 images) has the accuracy of 87.25 ± 2.24% and dataset B (528 graphs distributed equally) has the accuracy of 86.63% ± 5.78%, which is promising.

**Fig. 6:**
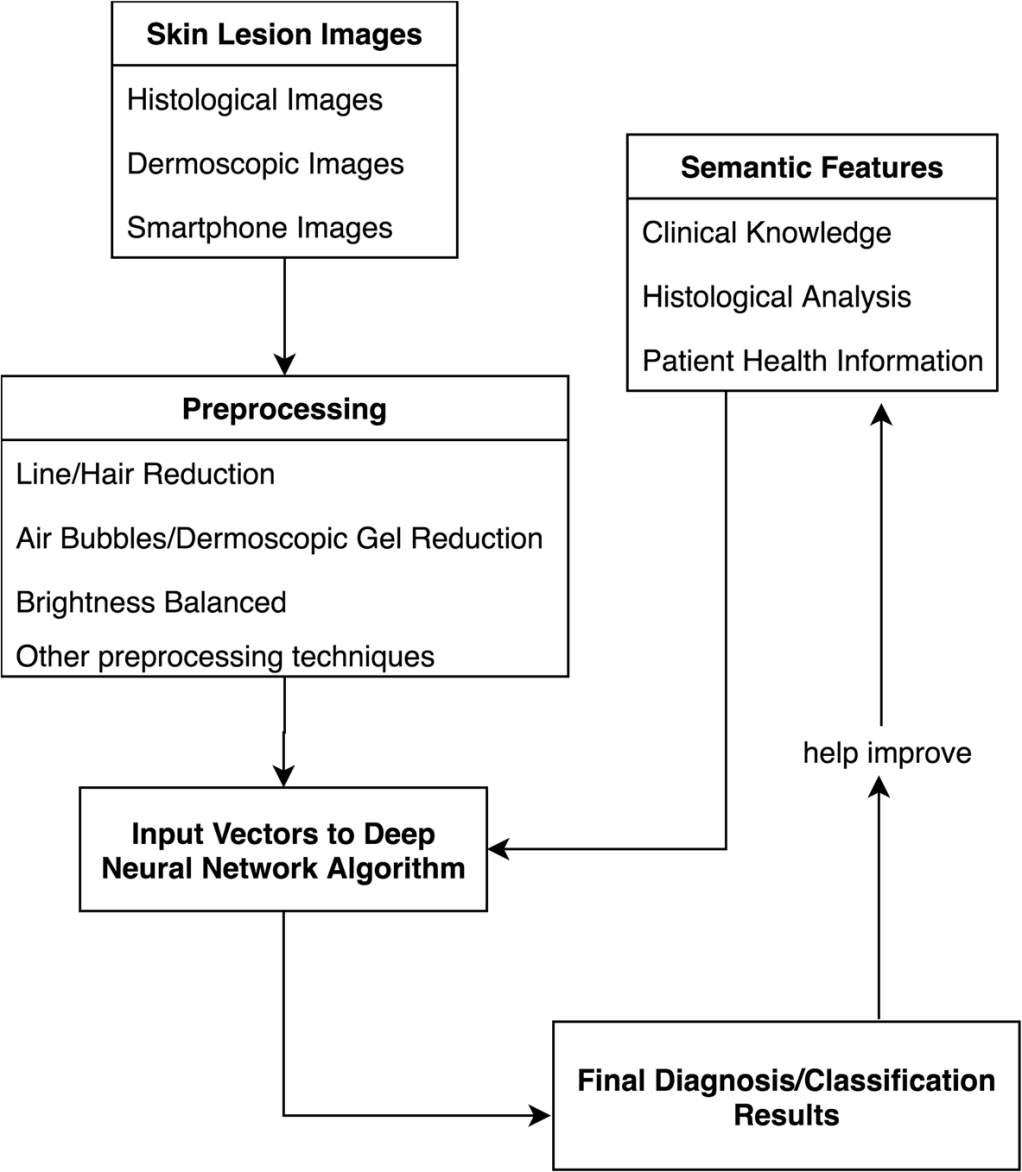
Human intelligence joint with computer aided system

A team of informaticians and dermatologists conducted a result analysis, especially for the misclassified images. Based on the result, we generated a hierarchical semantic structure (Fig. 6) to represent classification/diagnosis scenarios to further improve the algorithm to facilitate computer-aided decision support.

In a subsequent study, we would like to explore the method of integrating more human knowledge into our algorithm based on the result analysis. We will also make the system more extensive and scalable by handling larger datasets and more diseases and image types.

## Abbreviations

BCC: Basal cell carcinoma
CNN: Convolutional Neural Network
SK: Seborrheic keratosis
